# The linear relationship between systolic pulmonary artery pressure and mean pulmonary artery pressure is maintained regardless of autonomic or rhythm disturbances

**DOI:** 10.1186/s12931-016-0350-7

**Published:** 2016-03-31

**Authors:** Frédéric Vanden Eynden, Judith Racapé, Jame Vincent, Jean-Luc Vachiéry, Thierry Bové, Guido Van Nooten

**Affiliations:** Department of Cardiac Surgery, Université Libre de Bruxelles, Hôpital Académique Erasme, 808 Route de Lennick, B-1070 Brussels, Belgium; Cardiology, Université Libre de Bruxelles, Hôpital Académique Erasme, Brussels, Belgium; Anesthesiology Departments, Université Libre de Bruxelles, Hôpital Académique Erasme, Brussels, Belgium; Research Center of Biostatistics, Epidemiology and Clinical Research, School of Public Health, Université Libre de Bruxelles, Brussels, Belgium; Laboratory of Experimental Cardiac Surgery, University Hospital of Gent, Gent, Belgium

**Keywords:** Pulmonary circulation physiopathology, Pulmonary artery pressure, Transplantation

## Abstract

**Background:**

In the pulmonary circulation, there is a linear relationship between systolic pulmonary arterial pressure (SPAP) and mean pulmonary arterial pressure (MPAP). The aim of this study was to determine the passive or active nature of this mechanism by exploring the relationship in patients with and without autonomic rhythm control of the heart and pulmonary circulation.

**Methods:**

Pulmonary arterial pressure recordings from non-transplanted patients and patients with heart transplants or double lung transplants were retrospectively reviewed. The relationships between systolic, diastolic, and mean pulmonary arterial pressures were explored.

**Results:**

A linear relationship was observed between the SPAP and MPAP, whether patients were paced (MPAP = 0.56 SPAP + 3.86 mmHg, *r*^*2*^ = 0.889), treated with inotropes (MPAP = 0.55 SPAP + 5.52 mmHg, *r*^*2*^ = 0.947) or pulmonary vasodilators (MPAP = 0.58 SPAP + 2.41 mmHg, *r*^*2*^ = 0.927), were exercising (MPAP = 0.61 SPAP + 1.18 mmHg, *r*^*2*^ = 0.967), had a heart transplant (MPAP = 0.66 SPAP +0.87 mmHg, *r*^*2*^ = 0.849), a double lung transplant (MPAP = 0.7 SPAP +0.48 mmHg, *r*^*2*^ = 0.915), or no intervention (MPAP = 0.59 SPAP +1.75 mmHg, *r*^*2*^ = 0.937).

**Conclusion:**

We demonstrate that the linear relationship between SPAP and MPAP remains in several situations. Therefore, we conclude that the underlying mechanism is a passive consequence of the elastic properties of the cardiopulmonary unit.

## Background

The dynamics of the flow of blood generated by the ventricle and the characteristics of the effluent arterial bed determine the shape of the blood pressure curve. In the systemic circulation, increased pulse pressure has long been recognized as resulting from arterial wall stiffening and is related to increased mortality [1, 2]. While trying to use pulse pressure in the pulmonary circulation as a diagnostic tool for various etiologies of pulmonary hypertension, several investigators have identified a linear relationship between systolic (SPAP) and mean (MPAP) pulmonary artery pressures [3–6].

Based on the work of Saouti, Stergiopulos [7, 8], and Lankhaar [9], this observation has been explained by the inverse relationship between resistance and compliance, maintaining a constant product.

Though this linear dependency was seen throughout various etiologies of pulmonary hypertension, it has not been clearly demonstrated that the linearity is a passive consequence of a monotonous system or an active mechanism adapting the stroke volume, such as a chronotrope or vasomotor effect.

In this study, we explore the effect of autonomic afferences to the heart and lungs on the SPAP/MPAP relationship by investigating patients without those afferents; namely, lung and heart transplant patients. The effect of rhythm control on the linearity of the relationship was also considered and patients without intrinsic controls such as those with atrial fibrillation or ventricular pacing were considered to test our hypothesis that the linear relationship between SPAP and MPAP is passive and not the result of a rhythm adaptation or a vasomotor autonomic reflex.

## Methods

This retrospective study was approved by the Hospital Research Ethics Board (number P2013/184), which waived the need for patient consent for the anonymous use of their data.

Patients were divided into three categories; non-transplanted patients, heart transplant patients, and pulmonary transplant patients.

### Non-transplanted patients

All indwelling catheter procedures performed in the pulmonary hypertension and heart failure clinic between 1990 and 2012 were reviewed. Patients were included whether they had a diagnostic or follow-up procedure (assessment of treatment efficiency or clinical deterioration). Patients who had a pulmonary catheter inserted for monitoring purposes were excluded. A total of 514 patients were included in this study.

Clinically relevant information included age and gender. In the baseline group, we reviewed the charts of patients to classify them as having normal pulmonary pressures or having pulmonary hypertension, which was further categorized according to the World Health Organization classification [10]. Patients without intrinsic rhythm control were identified by reviewing the charts for atrial fibrillation or pacemaker implantation and confirmed by irregular rhythm on electrocardiograms or by pacemaker stimulation during the pressure measurements. We excluded 21 patients based on inconclusive charts. From the remaining 493 patients, we identified 52 patients with rhythm disturbances.

According to the conditions in which the procedures were performed, patients were divided into five sub-categories: baseline measures, during exercise testing, or while receiving pulmonary vasodilators, nitric oxide (NO) inhalation, or inotropes.

When repeated measures on the same patient where done under the same conditions, only one measure was used to avoid statistical inference by repeated measures. When either the systolic or diastolic value was missing, the record was excluded.

A total of 132 measures performed at rest during inhalation of 20 parts per million (ppm) NO were used for analysis. Patients were divided into groups of responders and non-responders if their MPAP diminished more than 20 % or not according to Sitbon et al. [11].

Twenty nine measures were considered for analysis in patients being perfused with inotropes (1, 16, 5, 1, 2, and 2 patients that were treated with 12, 10, 8, 6, 4, and 2 mg · kg · min of dobutamine, respectively; one patient treated with 0.5 mg milrinone and one patient treated with 10 mg · kg min enoximone).

Some right catheters were inserted and the patients were asked to exercise. Although different workloads were used during the same sessions, only one workload was considered per patient. Workloads varied from 10 to 75 W.

**Fig. 1:**
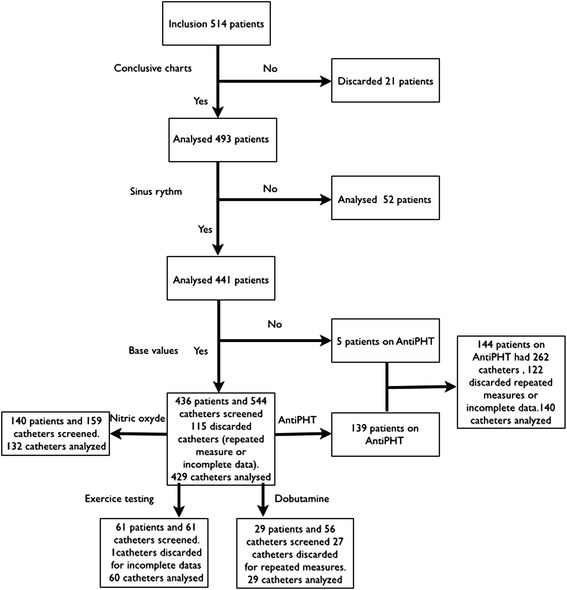
Flowchart of catheter allocation and analysis in the non-transplanted patient group

Figure 1 represents all screening procedures distributed into the different sub-categories.

### Heart transplant patients

From November 1981 to December 2012, a total of 524 heart transplants were performed in 512 patients at our institution. All of these patients were reviewed and those who benefitted from a right heart catheterization with pressure measurements were included in the study.

The population was split into two categories; patients who underwent a right heart catheterization during the first 2 years post-transplant, and patients who were catheterized after the first 2 years. This was done in order to explore the eventual effect of cardiac re-innervation. Eighty-three patients had 108 right heart catheterizations performed within 2 years of heart transplantation, and 83 records were maintained in this study to prevent any statistical inference of repeated measures. Sixty-eight patients had 106 right heart catheterizations done more than 2 years after heart transplantation, and 68 records were evaluated in this study.

### Pulmonary transplant patients

From January 2007 to December 2012, 178 patients underwent double lung transplantations. All of these patients were included in this study. Pressures were recorded by means of a Swan-Ganz catheter inserted before the pulmonary transplant and repositioned in the main pulmonary artery after the procedure. Measurements were made 24 h after the patient reached the intensive care unit to prevent any recordings that occurred during a hemodynamically unstable period. Two patients died within 24 h and 11 patients had their pulmonary artery catheters retrieved or ill-positioned, yielding unreliable right heart catheter records.

All of the recording was done using Swan-Ganz catheters (Edwards Lifescience, Evaston, CA, USA), which were inserted into the jugular vein using the Seldinger technique. The tip of the catheter was positioned in the pulmonary artery with pressure guidance or fluoroscopy if needed. Peak systolic and end diastolic pulmonary artery pressures were measured, and mean pulmonary artery pressure was calculated using software embedded in the measurement instruments. When more than one measurement was made under the same conditions during the same procedure, only the first one was taken into account. Wedge pressure and central venous pressure were also recorded.

### Statistical analysis

Simple linear regression on each set of different measurements was used to generate prediction equations relating MPAP, diastolic pulmonary artery pressure (DPAP), and SPAP; goodness of fit was assessed with R^2^.

Comparison of slopes and intercepts was performed according to Armitage [12], and *p* < 0.05 was considered statistically significant.

The software, MedCalc Statistical Software version 14.12.0 (MedCalc Software bvba, Ostend, Belgium; http://www.medcalc.org; 2014)) was used for analysis.

## Results

**Fig. 2:**
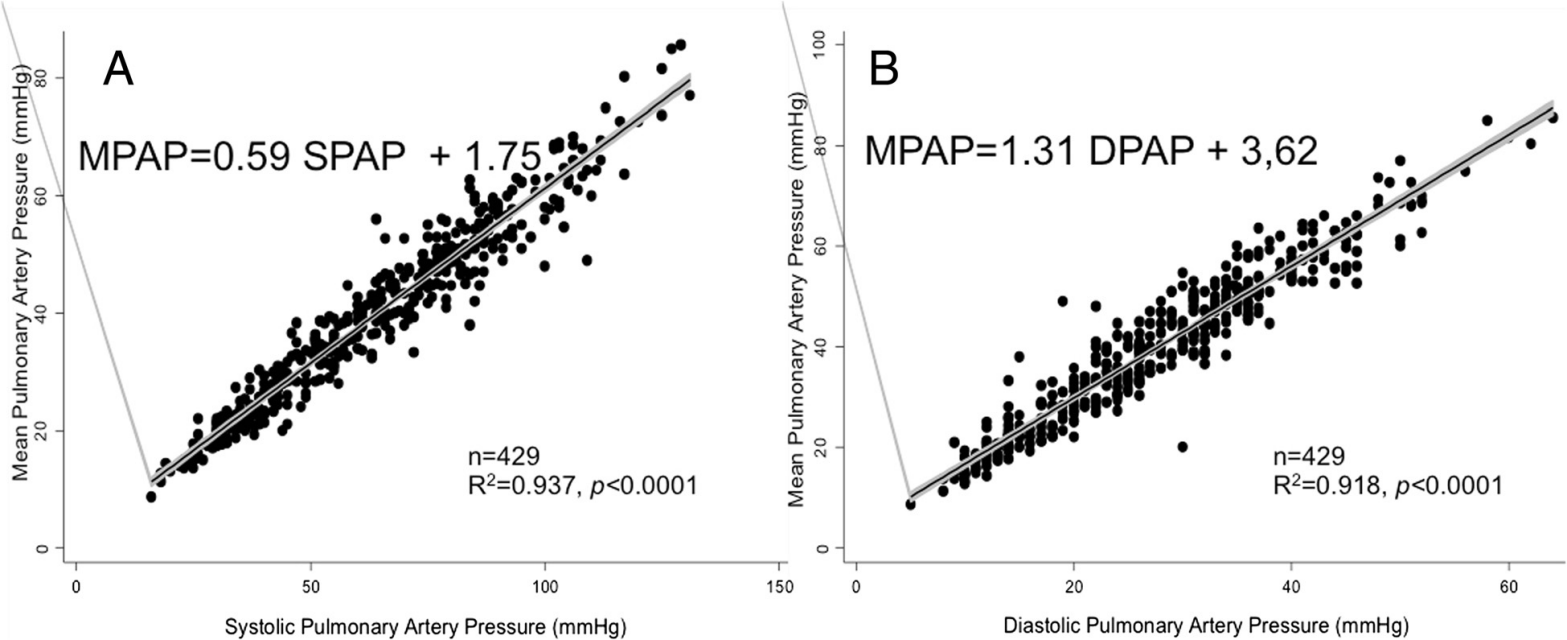
Linear relationship between pulse pressures in non-transplanted patients. **a** Linear relationship between MPAP and SPAP. **b** Linear relationship between MPAP and DPAP

**Fig. 3:**
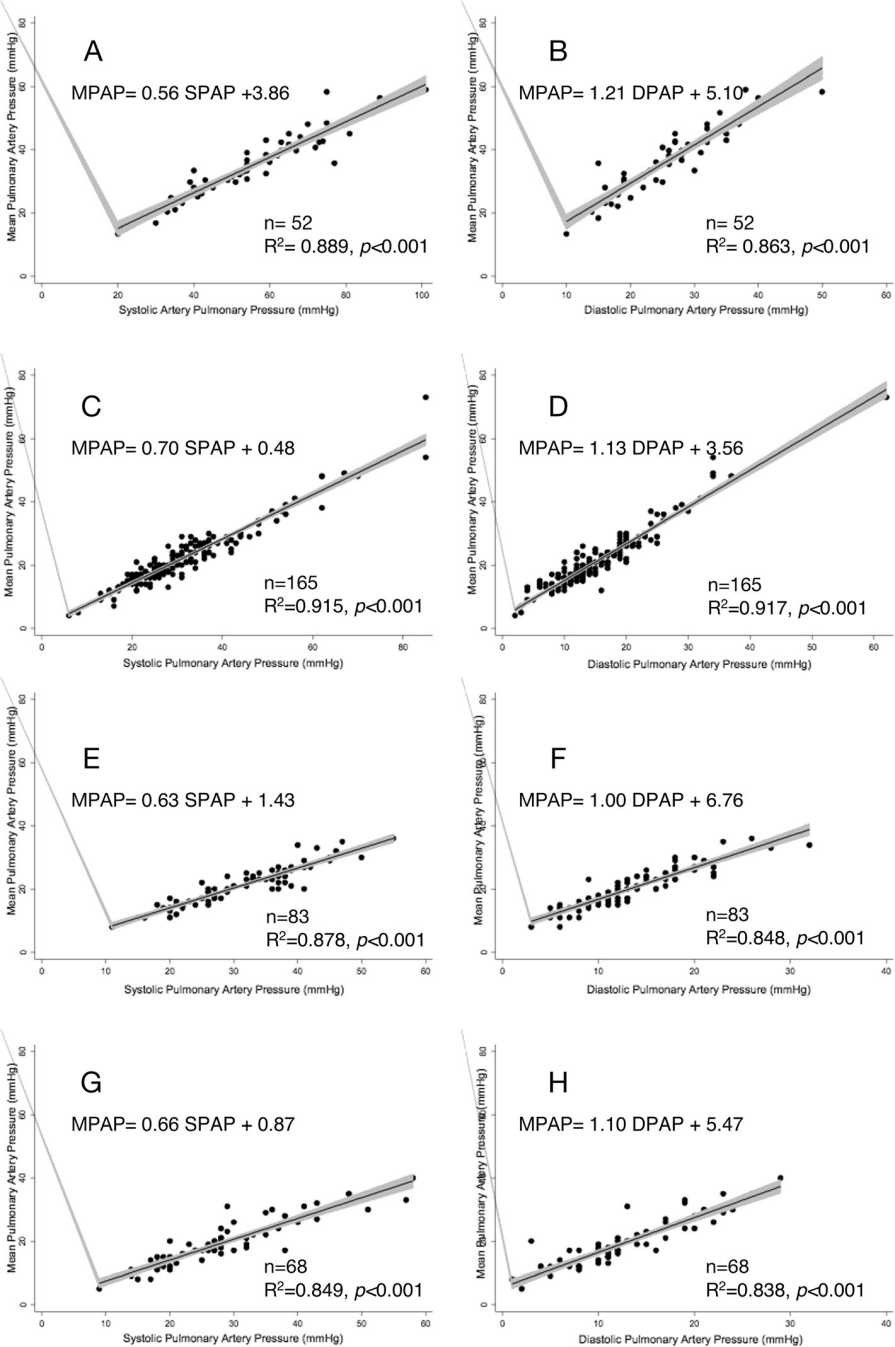
Linear relationship between MPAP and SPAP or MPAP and DPAP. **a**, **b** Non-transplanted patients who were not in sinus rhythm, **c**, **d** lung transplant patients, **e**, **f** heart transplant patients in the first 2 years after transplantation, and **g**, **h** heart transplant patients more than 2 years after transplantation

Baseline measurements in patients in sinus rhythm are presented in Fig. 2. MPAP and SPAP were linearly related according to the following equation: MPAP = 0.59 SPAP + 1.75 mmHg (*r*^*2*^ = 0.937; 429 patients). MPAP and DPAP were linearly related according to the following equation: MPAP = 1.31 DPAP + 3.62 (*r*^*2*^ = 0.918; 429 patients). In Table 1, patients in the baseline group are divided between those with and without (normal) pulmonary hypertension. Patients with pulmonary hypertension were further subdivided according to the etiology of their disease. The relationships between MPAP, SPAP, and DPAP are presented; the differences between the coefficients of the different slopes were not statistically significant. A linear relationship was observed between MPAP and SPAP for patients who were not in sinus rhythm (MPAP = 0.56 SPAP + 3.86, *r*^*2*^ = 0.889; 52 patients), patients who underwent double lung transplant (MPAP = 0.7, SPAP +0.48, *r*^*2*^ = 0.915; 165 patients), patients less than 2 years after a heart transplantation (MPAP = 0.63 SPAP +1.43, *r*^*2*^ = 0.878; 83 patients), and patients more than 2 years after heart transplant (MPAP = 0.66 SPAP + 0.87, *r*^*2*^ = 0.849; 68 patients; Fig. 3). Similarly, a linear relationship between MPAP and DPAP was observed (Fig. 3).

**Table 1:** Baseline group classified according to pathology

Group		Number of patients		MPAP	SD	Systolic Regression	R^2^	Diastolic Regression	R^2^
Normal		83		19	3.6	0.50 SPAP + 3.36	0.777	1.11DPAP + 4.00	0.976
Group 1		135		50	12,8	0.58 SPAP + 3.52	0.995	1.16DPAP + 9.68	0.995
	*IPAH*		*68*	51	11,2	0.60 SPAP + 2.35	0.838	1.10DPAP + 11.6	0.878
	*Medication induced PAH*		*10*	51	13,9	0.61 SPAP + 0.02	0.991	1.44DPAP+ 0.43	0.989
	*Cirrhosis*		*15*	52	12,5	0.60 SPAP −0.06	0.990	1.45DPAP + 1.07	0.986
	*Congenital*		*13*	57	16,8	0.61 SPAP −1.03	0.986	1.44DPAP + 1.50	0.976
	*Systemic sclerosis*		*22*	44	12,5	0.61 SPAP + 1.65	0.966	1.10DPAP + 4.07	0.939
	*HIV*		*2*	62	^a^				
	*Hereditary*		*1*	49	^a^				
	*schistosomiasis*		*1*	50	^a^				
	*veno-occlusive disease*		*3*	47	^a^				
Group 2		109		38	10,8	0.59 SPAP + 3.57	0.877	1.25 DPAP + 3.25	0.867
Group 3		26		39	9,4	0.54 SPAP+ 5.62	0.908	1.31 DPAP + 1.75	0.877
Group 4		63		44	10.4	0.53 SPAP + 3.93	0.789	1.12 DPAP + 11.50	0.987
Group 5		12		44	11,04	0.53 SPAP + 6.38	0.976	1.45 DPAP-0.20	0.856
Unclassifed		1		43	^a^	^a^	^a^	^a^	^a^

*IPAH* idiopathic pulmonary arterial hypertension, *MPAP* mean pulmonary arterial pressure, *SPAP* systolic pulmonary arterial pressure, *DPAP* diastolic pulmonary arterial pressure, *SD* standart deviation

MPAP is expressed in mmHg

^a^where calculations are not applicable

**Fig. 4:**
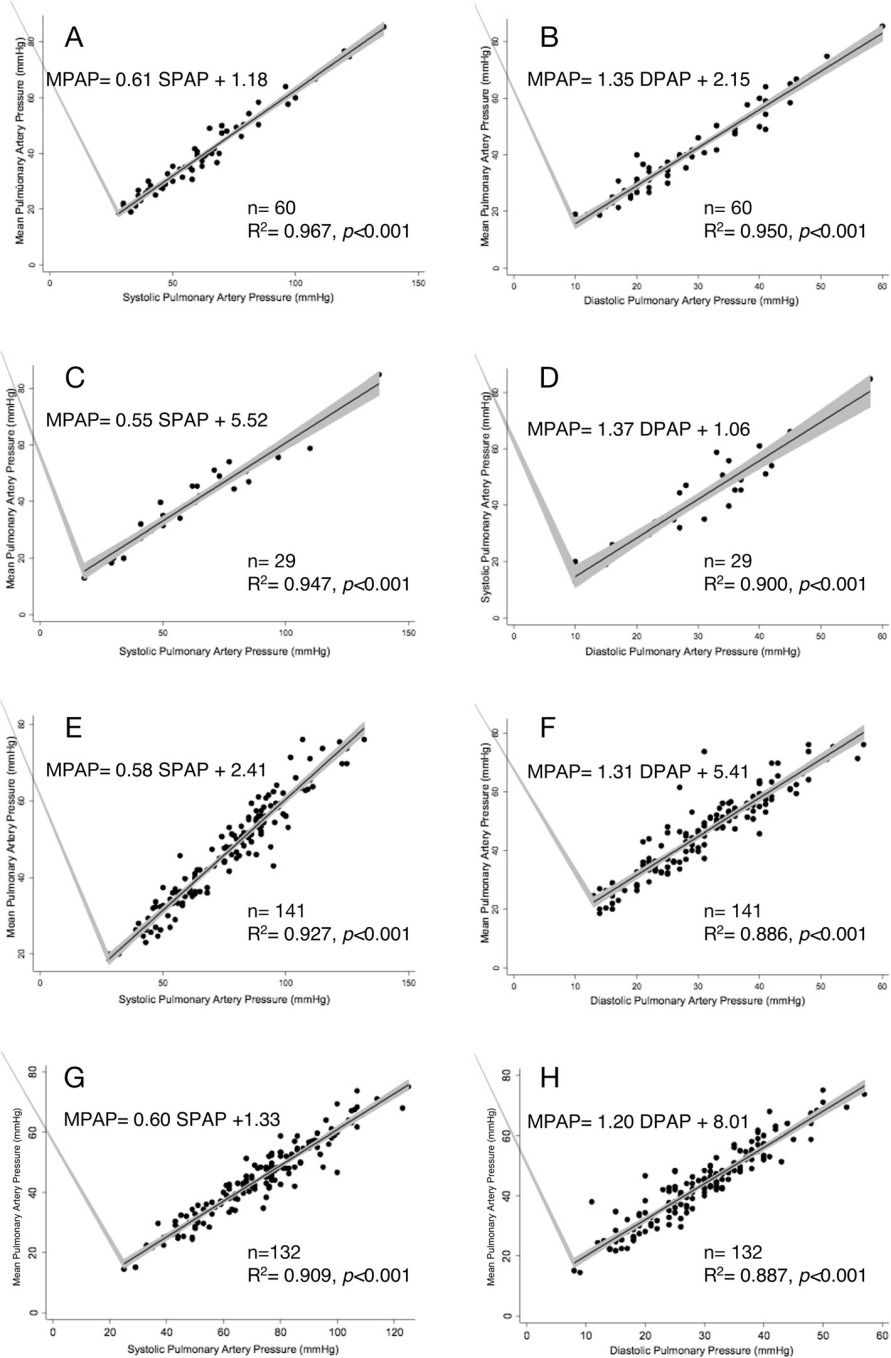
Linear relationship between MPAP and SPAP or MPAP and DPAP. **a**, **b** In sinus rhythm at exercise; **c**, **d** while given inotropes; **e**, **f** on pulmonary vasodilators; **g**, **h** with NO inhalation

A linear relationship was also observed when patients were exercising (MPAP = 0.61SPAP + 1.18, *r*^*2*^ = 0.967; MPAP = 1.35 DPAP +2.15, *r*^*2*^ = 0.950, 60 patients), when patients were given inotropes (MPAP = 0.55 SPAP +5.52, *r*^*2*^ = 0.947; MPAP = 1.37 DPAP + 1.06, *r*^*2*^ = 0.9, 29 patients), or when they were treated with pulmonary vasodilators (MPAP = 0.58 SPAP +2.41, *r*^*2*^ = 0.927, MPAP = 1.31 DPAP + 5.41, *r*^*2*^ = 0.8660, 141 patients; Fig. 4).

In the group inhaling NO, 19 patients (14.3 %) were identified as responders (basal MPAP = 49 ± 14 mmHg; with 20 ppm NO: MPAP = 33 ± 11 mmHg), and 113 patients were identified as non responders (basal MPAP = 49 ± 11 mmHg; with 20 ppm NO: MPAP = 46 ± 10 mmHg). The relationship was also linear in the responder (MPAP = 0.58 SPAP +0.71, *r*^*2*^ = 0.895; MPAP = 1.221 DPAP = +6.013, *r*^*2*^ = 0.893) and non-responder (MPAP = 0.59 SPAP = +1.97, *r*^*2*^ = 0.89; MPAP = 1.135 DPAP = +10.426, *r*^*2*^ = 0.842) groups (Fig. 4g and h).

The slope and intercept of the equation of every subgroup was compared to the baseline equation of the patients in sinus rhythm (MPAP = 0.59 SPAP + 1.75). None of the differences observed were statistically significant except for the equation describing the relationship in patients after double lung transplantation (MPAP = 0.7 SPAP + 0.48).

## Discussion

Similar to what other studies have already shown in smaller cohorts of patients, our study explored the linear relationship between the SPAP and MPAP [3–6]. We looked at the effects of various medical interventions such as pulmonary vasodilators and inotropes and the influence of more complex physiological inferences such as exercise.

Our baseline measurements had coefficients of the linear regression (0.59 SPAP + 1.75) that were remarkably similar to those identified by Chemla with high fidelity catheters (0.61SPAP + 2). This empirical formula also is the most accurate compared to other modalities of evaluating MPAP [13].

This linear relationship at rest was also observed in our study of patients treated with pulmonary vasodilators or dobutamine and even the complex physiological situation of the stress test, when there is a mixed inotropic and chronotopic effect on the right ventricle and a lusitropic effect on the left ventricle [14]. Whether or not patients were responders to NO, a pulmonary vasodilator widely used in pulmonary hypertension or acute respiratory distress syndrome that has shown little clinical benefit in clinical trials [15], had no influence on the linear relationship (Fig. 4g and h).

**Fig. 5:**
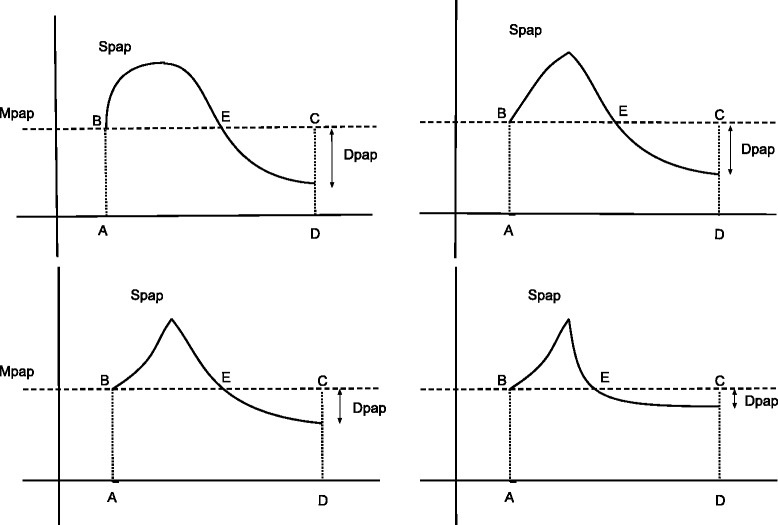
Geometric limitation of the DPAP. Four fictive pulmonary pressure waves are illustrated sharing the same MPAP and SPAP. For a given period, if the MPAP are equal, then the areas **a**, **b**, **c**, and **d** are the same in all cases. The area B, SPAP, E and E, DPAP, and C have to be equal. Changing the area of A,SPAP, and E is geometrically limited and include the possible values of DPAP

The physiological implication is significant. The MPAP is the pressure observed if the area under the pressure curve is reduced to a rectangle, which means that the area delimited above and below the MPAP curve have to be equal. Through the various observations showing that SPAP and MPAP are linearly related, we must admit that SPAP is fixed when MPAP is defined. The area above the MPAP curve is relatively restricted since SPAP is fixed. Variations in the area are possible due to curve alterations or timing of the MPAP line crossing (Fig. 5). The area under the MPAP curve adapts, thanks to the DPAP values that are as a result confined in a relatively narrow range. This scenario is valued by our observations since DPAP and MPAP are linearly related, although with an r coefficient that is systematically lower than the one observed for the SPAP/MPAP relationship.

**Fig. 6:**
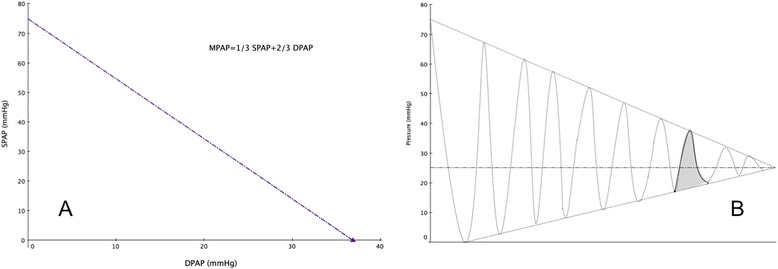
Theoretical and effective values of the pulse pressure. All possible values for SPAP and DPAP allowed by the rule of thumb are represented along the line in (**a**), although only one pair would be physiologically observed as illustrated in (**b**)

From these geometric considerations, we can assume that despite the multiple values of pulse pressure that are obtainable from the classic rule of thumb (MPAP = 1/3SPAP + 2/3DPAP), only one can be physiologically possible (Fig. 6). This means that once SPAP is defined, pulse pressure is restricted to narrow values and would be of low diagnostic value in order to discriminate between various forms of pulmonary arterial hypertension. However, some caution is warranted concerning this theoretical reasoning since some authors have successfully relied on the pulse pressure for more elaborate purposes such as partitioning the resistance in chronic thromboembolic disease [16, 17], while others have failed to do so [18].

This remarkable property of the pulmonary circulation, which is quite different from its systemic counterpart, may have different explanations. The question remains whether its nature is active or passive. One might postulate that an autonomic feedback control affects the resistance of the pulmonary tree, inotropic properties of the right ventricle, and the cardiac rhythm in order to maintain the pulse pressure in its defined value around the MPAP. If this hypothesis were true, the linear relationship between MPAP and SPAP would disappear when the rate control disappears, such as in patients with atrial fibrillation or artificially paced hearts. This was not observed in our patients; the linear coefficient was quite consistent with the empirical formula (0.56SPAP + 3.86). Moreover, the crosstalk between the pulmonary circulation and the right heart is also not mediated through autonomic innervation of the heart, because the de-innervated transplanted hearts produced a similar pulse pressure pattern (0.63 SPAP + 1.43).

When the right heart is pumping blood to freshly transplanted lungs, the relationship is a little different. The pulse pressure is narrowed because not only does the SPAP tend to incline towards the MPAP (0.7SPAP + 0.48), but so does the DPAP (1.16DPAP + 3.58). Notably, the linear relationship stands despite the absence of residual autonomic afferents to the lungs, while only the coefficients are different. Therefore, three hypotheses should be investigated in the future. First, the discrepancy in the coefficient could be explained by the inherent imprecision of the fluid-filled catheters. Second, pressures are recorded at the tip of the catheter that lies in the pulmonary artery distal to the pulmonary anastomosis, wherein the elastic wall discontinuity may dampen the pressure wave. Finally, the concomitant change in pulmonary resistance and compliance caused by the lung transplantation could induce the deviation. Those changes are related to the structural changes introduced in the system by the new lungs and by the interruption of nervous afferents to the transplanted organs.

From the previous observations, we can postulate that the linear relationship between the MPAP and SPAP seems to be a monotonous phenomenon independent of any active control, which has been suggested by other authors emphasizing that the relationship is observed irrespective of the patient’s age, sex, heart rate, cardiac output, or pulmonary wedge pressure [5, 6], but it has not been reported in transplanted patients.

The pulmonary circulation serves a different purpose than the systemic circulation. The latter distributes blood to some vascular beds while shutting off others, whereas the pulmonary circulation tries to ensure an evenly distributed flow matching the ventilation areas. Therefore, its compliant compartment extends far into the periphery [19] and the already low basal resistance even decreases during exercise. The relationship between resistance and compliance has been postulated to be inversely related, and the product of both parameters (RC). This is because the time constant of the exponential decay function portraying the diastolic portion of the pulmonary pressure curve is considered constant. The constancy of RC has been shown in healthy patients [20] and those with diseased conditions [9], and even in single lung ventilation in the setting of thromboembolic hypertension [8]. The hyperbolic relationship between pulmonary vascular resistance and arterial compliance has been advocated as the reason for the proportional relationship between SPAP and MPAP [21]. The problem with computing values based on compliance is that it is far from obvious to correctly measure compliance [22]. Moreover, to our best knowledge all clinical studies done using the RC quotient have recorded compliance as the ratio of stroke volume over pulse pressure, which overestimates compliance compared to one done with the more sophisticated pulse pressure method [23]. Compliance could be extracted from the diastolic part of the cardiac cycle [24], but the diastolic portion of the pressure curve is bi-exponential rather than mono-exponential [25], and measurements based on the diastolic decay of the pressure curve should be more accurate for identifying various inflexion points. Therefore, extracting compliance via curve fitting requires techniques that provide better performance than fluid-filled catheters [26]. These technical and physiological considerations might explain why some authors have found discrepancies in calculating the RC constant despite a constant SPAP/MPAP ratio in different pathological states [27]. Aware of these technical pitfalls, we preferred not to elaborate on the RC time constant and instead to witness the passive phenomenon of pulse pressure confinement.

### Limitations

The main limitations of this study are due to its retrospective design and the heterogeneity of the settings in which the measurements were made. Lung transplant patients were measured in the intensive care unit, heart transplant patients in the cath-lab, and the other patients in the coronary care unit. Another limitation is that all measurements were made with fluid-filled catheters, which are commonly prone to a damping phenomenon, and they yield less accurate readings in comparison with high fidelity catheters. We tried to overcome these shortcomings by including a large number of patients, especially in the transplanted cohort.

## Conclusion

The linear proportionality between MPAP and SPAP in the pulmonary circulation has been reported extensively in both healthy and disease states. Such observations were further investigated in conditions yielding diverse physiological challenges including administration of inotropes or pulmonary vasodilators, or during exercise. The proportionality was preserved despite disturbances of the native cardiac rhythm or autonomic inference, suggesting the passive nature of the predisposing mechanism.

## Abbreviations

C: compliance
DPAP: diastolic pulmonary artery pressure
MPAP: mean pulmonary artery pressure
R: resistance
SPAP: systolic pulmonary artery pressure
